# T2-weighted MRI pulse sequences for imaging post-infarct edema in mice: comparison of spin echo and T2 preparation approaches

**DOI:** 10.1186/1532-429X-11-S1-O33

**Published:** 2009-01-28

**Authors:** Ronald J Beyers, Yaqin Xu, Frederick H Epstein, Brent A French

**Affiliations:** University of Virginia, Charlottesville, VA USA

**Keywords:** Cardiac Magnetic Resonance, Delayed Enhancement, Phase Encode Gradient, Gadolinium Delayed Enhancement, Cardiac Magnetic Resonance Sequence

## Introduction

An ongoing diagnostic challenge exists in reliably differentiating non-salvageable, acutely infarcted myocardium from surrounding stunned, yet viable, myocardium that defines the area at risk. T2w cardiac magnetic resonance (CMR) imaging has previously been used to image the edema characterizing the area-at-risk region in post myocardial infarcted (MI) canine, porcine and human hearts. Similar techniques would be valuable in basic research studies of MI in mice, where they might be used jointly with gadolinium delayed enhancement (DE) imaging to non-invasively define infarct size as "% area-at-risk" in transgenic/knock-out mice. However, the rapid murine heart rate presents challenges to T2w CMR application in mice. The typical T2w echo time of 40–60 ms needed for the detection of edema occupies a significant portion of the murine cardiac cycle (100–120 ms) with significant periods of blood flow and cardiac motion.

## Purpose

Develop an effective T2w CMR sequence for mice that exhibits high immunity to flow and tissue motion artifacts while maintaining sufficient and consistent signal-to-noise (SNR) and contrast-to-noise (CNR) performance.

## Methods

We developed two T2w sequences for murine CMR: a flow and motion desensitized spin-echo (SE) sequence and a T2 preparation (T2prep) sequence. The SE sequence employed a slice-selective excitation RF pulse and a thicker slice-selective refocusing RF pulse, and applied readout and phase encoding gradients after the refocusing pulse. The T2prep sequence employed non-selective MLEV-weighted composite RF pulses followed by a standard slice-selective gradient-echo readout. Each sequence was applied on an isoflurane-anesthetized mouse on Days 1 through 4 after reperfused MI induced by 60 min coronary occlusion, as described previously. Parameters for both sequences included TR = 1500 ms, TE = 40–60 ms, FOV = 25 × 25 mm, slice thickness = 1 mm, matrix = 128 × 128 and BW = 520 Hz/pixel. In addition, gadolinium-DTPA DE CMR was performed each day to define infarct location. All scans of each sequence were performed consecutively at four identical contiguous slice positions from mid-ventricular toward the apex. All scans were performed on a 7 T Bruker/Siemens ClinScan.

## Results

**Figure 1:**
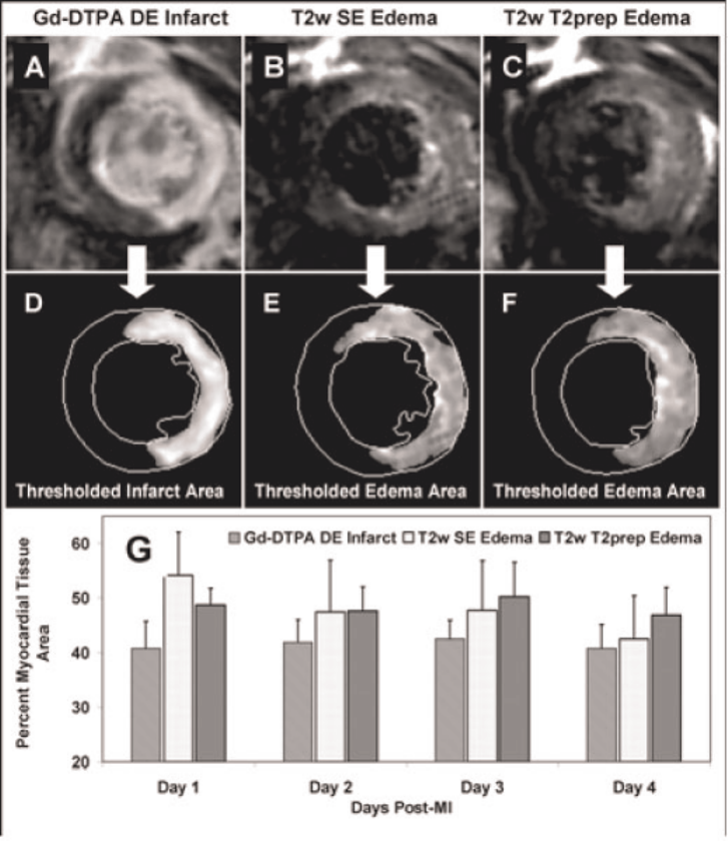
**Gd-enhanced mid-ventricular image of the mouse heart 1 day after MI, showing enhanced infarct region (A) with corresponding thresholded infarct region (D)**. Same slice T2w spin echo image (B) with corresponding thresholded edema (E). Same slice T2w T2prep image (C) with corresponding thresholded edema (F). Comparison of detected infarct and edema region areas over Days 1 through 4 post-MI for each imaging method.

Panels A through C (Fig. 1) show example sets of Gd-DTPA DE, T2w SE and T2w T2prep images (respectively) from the same LV slice position at Day 1 post-MI. Panels D through F show the respective hyperintense regions identified by threshold analysis performed after manual segmentation of the left ventricular (LV) myocardium. Threshold analysis for all images selected any pixels within the segmented myocardium with >2 standard deviation magnitude than a myocardial image sample chosen remote from the infarct area. Panel G tracks and compares the detected infarct and edema region areas over Days 1 through 4 post-MI for each imaging method. Both T2w sequences detected regions of edema that corresponded to or surrounded the infarct as determined by DE CMR. The T2w SE sequence gave a consistently higher SNR of 104 ± 6 (mean ± SEM) and CNR of 52 ± 3 over the T2prep SNR of 68 ± 4 and CNR of 34 ± 2, where CNR was measured between remote and threshold-selected myocardium. However, the SE sequence had a higher occurrence of flow and motion artifacts that degraded the consistency and accuracy of threshold selection.

## Conclusion

As shown (panel G), the T2prep yielded a consistently higher edema area percentage of 48.2 ± 2.5 compared to the infarct area percentage of 41.6 ± 1.8, which correlates well with previous canine and human studies. Meanwhile, the SE gave a slightly lower mean with higher variance edema area percentage of 47.0 ± 4.2 that was attributed to flow and motion artifacts. To our knowledge, this is the first study to demonstrate the feasibility of performing T2w CMR edema imaging in mice, which opens a variety of potential basic research applications investigating the role of individual genes in acute and chronic settings post-MI.

